# Protein supplementation in pediatric oncology: a systematic review revealing a lack of evidence and insights from other chronically and critically ill children

**DOI:** 10.3389/fped.2025.1724658

**Published:** 2025-12-10

**Authors:** Emma den Hartog, José van Tongeren, Elvira C. van Dalen, Mirjam van den Brink, Emma J. Verwaaijen, Wim J. E. Tissing

**Affiliations:** 1 Princess Máxima Center for Pediatric Oncology, Utrecht, Netherlands; 2 Department of Pediatric Oncology and Hematology, University of Groningen, Beatrix Children’s Hospital, University Medical Center Groningen, Groningen, Netherlands

**Keywords:** pediatric oncology, chronic illness, critical illness, protein supplementation, anthropometrics, body composition, physical fitness, adverse effects

## Abstract

**Background & aims:**

Exercise interventions may counteract adverse effects of childhood cancer (treatment) on anthropometrics, body composition and physical fitness. However, inadequate nutritional status and loss of muscle mass and strength might limit effectiveness. Protein supplementation might support muscle preservation and enhance the benefits of exercise but evidence on its effect in children with cancer is lacking. This review examined the potential effect of protein supplementation on anthropometrics, body composition, physical fitness and adverse effects in children with cancer from diagnosis until two years after end of treatment.

**Methods:**

PubMed/MEDLINE was searched for literature on childhood cancer and protein supplementation (14th of May 2025). Reports examining anthropometrics, body composition and/or physical fitness before and after the protein intervention and written in English were eligible. Another search was performed in chronically and critically ill children (14th of May 2025). Risk of bias was assessed using Cochrane Childhood Cancer criteria and for each outcome, we graded the quality of evidence using the Grading of Recommendations Assessment, Development and Evaluation methodology. Data were descriptively reported.

**Results:**

None of the studies in children with cancer could be included. Five studies were included with a total of 142 children (66 had cystic fibrosis, 6 Crohn's disease, 70 a prolonged pediatric intensive care unit stay). Protein supplementation interventions varied. All studies reported on anthropometrics and body composition. Four observational studies reported a significant improvement in at least one of the outcomes whereas one randomized controlled trial (RCT) found no significant changes. Of the three studies assessing physical fitness measurements, two observational studies reported a significant improvement in at least one of the outcomes while one RCT did not find a significant change. The three studies reporting on adverse effects were encouraging but this outcome deserves further investigation. Level of evidence was graded very low for all outcomes.

**Conclusions:**

Evidence on the potential of protein supplementation is scarce and not available in children with cancer. The limited evidence included in this review tentatively suggests that protein supplementation might aid in improving anthropometrics, body composition and physical fitness. Future controlled trials are needed to determine its feasibility and effectiveness in children with cancer.

## Introduction

The overall five-year survival of children with cancer nowadays exceeds 80% in high income countries (1). Despite improved survival rates, children with cancer frequently suffer from adverse health effects, including loss of muscle mass and strength. A recent review showed that they experience significant losses of skeletal muscle during intensive early treatment phases while simultaneously they gain fat mass, independent of cancer type (2). These effects might be caused by prolonged hospitalization, immobilization and reduced mobility and physical activity as a result of children's disease and the (catabolic) effects of its treatment (3–5). All of these might negatively impact children's anthropometrics, body composition and physical fitness and in turn decrease their functional independence, ability to participate in daily life activities and quality of life. Exercise interventions, including muscle strengthening training, during childhood cancer treatment might help to prevent muscle wasting and weakness by supporting physical function, mobility, muscle strength and reducing pain (5, 6). Consequently, exercise has the potential to mitigate the adverse health effects of childhood cancer and its treatment on anthropometrics, body composition and physical fitness.

In addition to an increased risk of reduced physical fitness, children with cancer are vulnerable to developing a suboptimal nutritional status (7–9). They might have a poor dietary intake and experience feeding difficulties caused by treatment-related symptoms such as nausea, reduced appetite and changes in smell and taste (8). A prospective cohort study of 115 children diagnosed with cancer found that around one third had inadequate protein intake according to their individual age-matched requirements at the beginning of treatment and after twelve months (10). However, the optimal protein requirement for children with cancer to support physiological functions in line with their sex, age and disease remains unknown. Children with cancer might have higher protein requirements compared to healthy children to support recovery from their disease and its treatment as they might need to compensate for muscle wasting and loss of muscle strength (11) and/or have an enhanced metabolic rate (12). Appropriate protein intake in line with their individual protein requirements might benefit their ability to engage in exercise training and experience its advantages (13).

Although the advantages of exercise during childhood cancer treatment on anthropometrics, body composition and physical fitness, including improved muscle strength (14, 15), are recognized, literature about the effect of exercise interventions in children with a suboptimal nutritional status is lacking. Inadequate nutritional status and treatment-related loss of muscle mass and strength might limit its effectiveness. Protein supplementation might support muscle preservation and enhance the benefits of exercise but evidence on the effect of protein supplementation in children with cancer is lacking. It holds promise to enhance anthropometrics, body composition and physical fitness and subsequently contribute to enhanced quality of life of these children. In adult cancer patients, protein supplementation led to significant improvements in fat-free mass index, body weight and muscle strength (16). Moreover, the addition of a protein supplement on top of an exercise intervention resulted in improved muscle strength compared to the exercise intervention alone in adult cancer patients (17). In contrast, in adult survivors of childhood cancer, a protein supplement combined with an exercise intervention did not result in changes in lean mass and muscle strength compared with a placebo and the exercise intervention (18). In children with cancer, there is no agreement on the effect of protein supplementation on anthropometrics, body composition and physical fitness during treatment.

Therefore, our aim was to systematically review the literature on the potential effect of protein supplementation on anthropometrics, body composition and physical fitness as well as possible associated adverse effects in children with cancer from diagnosis until two years after end of treatment. Moreover, we aimed to identify evidence in other chronically and critically ill children with comparable catabolic or treatment-related burden.

## Material & methods

The methodology was based on the Preferred Reporting Items for Systematic Reviews and Meta-Analysis (PRISMA) guidelines (19).

### Search strategy and study selection

PubMed/MEDLINE was searched for literature assessing the effect of protein supplementation in childhood cancer patients. The search strategy is presented in Supplementary material 1. A second search was performed in other chronically and critically ill children as there was a paucity of data in children with cancer. The search strategy of this second search is presented in Supplementary material 2. Both search strategies used a combination of controlled vocabulary and text words. Duplicates were manually removed and title/abstract and full text screening were performed in Rayyan (20) by two independent reviewers (EdH, JvT). Conflicts were collectively resolved and in case consensus was not reached, the project team was consulted. Snowballing of reports selected for full text screening and related reviews on similar topics was performed by screening reference lists to identify additional studies not detected by the PubMed/MEDLINE searches.

### Eligibility criteria

The aim of this review was to capture all available evidence on the potential effects of protein supplementation on anthropometrics, body composition, physical fitness and possible associated adverse effects in pediatric populations with comparable catabolic or treatment-related burden. Therefore, we used the following inclusion criteria. Reports investigating the effect of protein supplementation on anthropometrics, body composition and physical fitness and on possible associated adverse effects in children diagnosed with any type of childhood cancer (aged 0 to 21 years at time of diagnosis) from diagnosis until two years after end of treatment were included. Also, other chronically and critically ill children aged 0 to 21 years during active illness and who received extra protein were eligible. For this review, we included both children with the following chronic health conditions: cystic fibrosis (CF), inflammatory bowel disease (IBD), bronchopulmonary dysplasia, cardiological diseases, cerebral palsy or acquired brain injury and critically ill children, defined as those admitted to the pediatric intensive care unit (PICU).

All definitions for anthropometrics, body composition, physical fitness and adverse effects as used in the included studies were eligible. Outcomes including nutritional intake and protein metabolism were not considered as these were beyond the scope of this review.

Reports comparing results of an intervention group receiving protein supplementation with a control group receiving standard of care as well as reports revealing pre- and post-intervention results in patients receiving a protein intervention were eligible. Any standard of care treatment described in the included studies was eligible and the only factor differing between the intervention and control groups might have been protein supplementation.

Proteins might have been supplemented in whatever way possible (e.g., dietary intake, supplements, tube feeding) and every additional protein intake was considered. Moreover, interventions not merely including protein (e.g., a protein-energy supplement in which proteins might be combined with other substances, such as carbohydrates) were also eligible.

Reports including both children and adults were only eligible if results were reported for children separately or when the majority of the patients were children (i.e., more than 90% are 21 years or younger at diagnosis). If a portion of the children did not receive extra protein, the report was only eligible when separate results for children who received extra protein were available. We did not use any limitation in publication period.

Reports with a historical control group were not included as more factors influencing anthropometrics, body composition, physical fitness and/or adverse effects rather than protein intake alone might have changed over time. Moreover, narrative reviews and reports describing results in animals, *in vitro*, in another language than English or with no full text available were excluded. Reports describing survivors of childhood cancer (i.e., more than two years after end of treatment) were also not included as this review aimed to investigate the effect of protein supplementation from diagnosis until shortly after end of treatment. More than two years after end of treatment, most of the direct effects of the disease and its treatment on physical fitness will have changed into possible late effects, thereby maybe encompassing a different patient population. Reports were excluded if they involved children with developmental disorders, such as Down syndrome, as well as those involving children with severe burns, metabolic diseases or food allergies, children born preterm or with very low birth weight, those treated in the Neonatal Intensive Care Unit and neonates.

### Data extraction and risk of bias assessment

Data extraction was performed by one reviewer (EdH) and verified by a second reviewer (JvT). The risk of bias of included reports was independently rated by two reviewers (EdH, JvT). The results of the assessments were discussed and potential discrepancies between the two reviewers were solved by reaching consensus. For observational reports, we used the Cochrane Childhood Cancer Risk of bias assessment criteria which were based on previously described checklists according to evidence-based medicine criteria (21, 22). A separate risk of bias tool was used for (randomized) controlled trials (23). Risk of bias criteria are presented in Supplementary material 3.

### Data analysis

As pooling of the results was not valid (based on heterogeneity of studies regarding study design, patients, interventions and outcomes), we provided descriptive results. For each outcome, we graded the quality of the evidence using the Grading of Recommendations Assessment, Development and Evaluation (GRADE) methodology (24). The baseline score, based on the type of study design, was 4 for randomized controlled trials (RCTs) and 2 for observational studies.

## Results

### Results of the search

**Figure 1:**
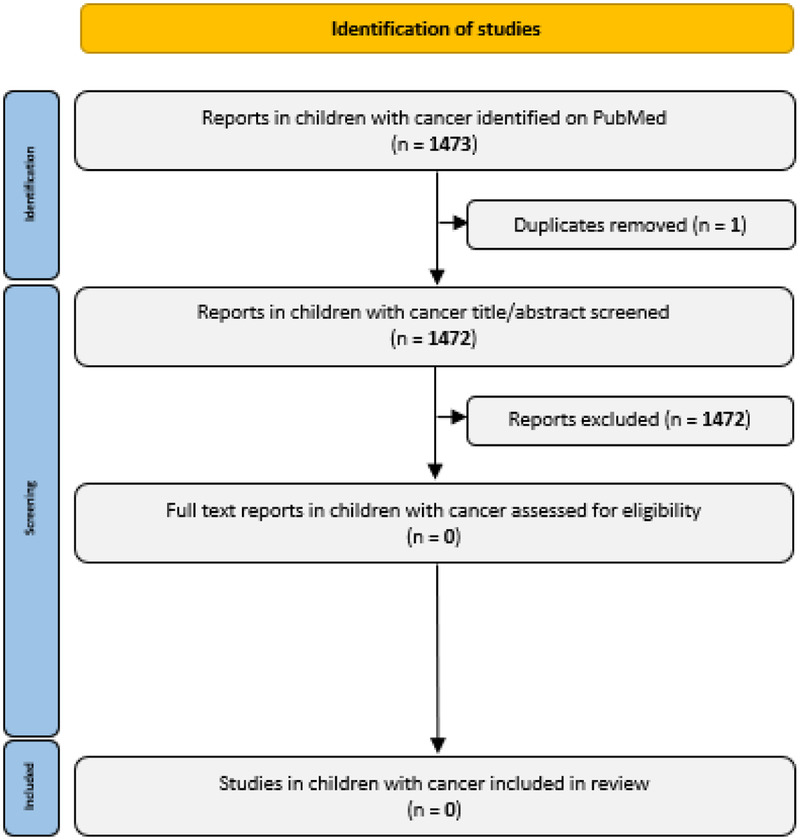
PRISMA flow diagram of study selection in children with cancer.

**Figure 2:**
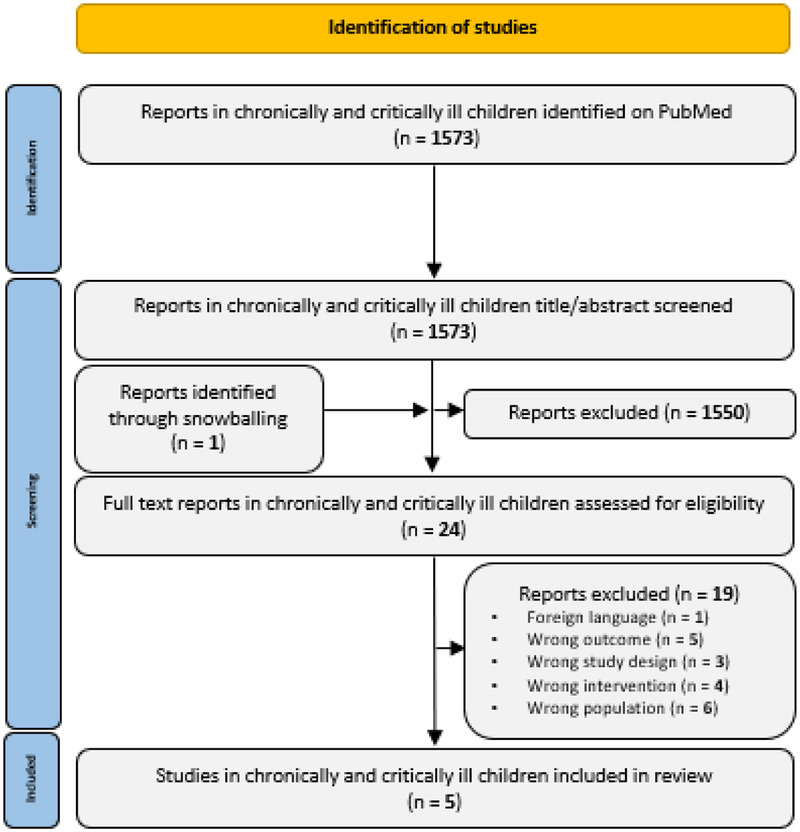
PRISMA flow diagram of study selection in chronically and critically ill children.

The PubMed/MEDLINE searches were run on May 14, 2025. After removal of duplicates, 1,472 reports were eligible for title/abstract screening for the search in children with cancer (Figure 1). None of these reports were eligible for full text screening. Another 1,573 reports were eligible for title/abstract screening after running the search in children with chronic health conditions or critical illness (Figure 2). After the addition of one report through snowballing, 24 reports were eligible for full text screening. Five of these were eligible for inclusion in this review (25–29).

### Study characteristics

One of the included studies was a RCT (28), while the other four were observational studies using pre- and post-intervention measurements (25–27, 29). One study evaluated the effect of pressurized whey (26), the other studies assessed the effect of protein and energy supplements (27–29) or a protein and energy-enriched (PE) formula (25). Three studies included children diagnosed with CF (26, 28, 29), one study included children with Crohn's disease (CD) (27) and in one study children had a prolonged PICU stay (25). More detailed information regarding the included studies is reported in Supplementary material 4. A summary of the evidence per outcome (i.e., anthropometric and body composition measurements, physical fitness measurements and intervention-related adverse effects) is shown in Tables 1–3 and elaborated on in the text below. A summary of the effects of the interventions on all outcomes is shown in Table 4.

**Table 1:** Summary of evidence on anthropometric and body composition measurements (*n* = 5 studies).

Study (author; year)	Study design	Patients (disease; number; age; gender; follow-up duration)	Intervention [type of supplement; dose; way of supplementation; frequency; duration; timing; standard of care (for randomized controlled trials only)]	Main results	Risk of bias
Lands et al., 2010 (26)	Prospective intervention cohort study	Cystic fibrosis;	Pressurized whey;	Weight Δ weight (g) mean increase 480 (SD 832), *p* > 0.1 Lean body mass Δ lean body mass (g) mean increase 322 (SD 736), NS Body fat percentage *Δ* body fat percentage (%) mean increase 0.5 (SD 2.25), NS BMI Δ BMI Z-score mean −0.69 pretreatment vs. mean −0.52 posttreatment, mean increase 0.16 (SD 0.206), *p* < 0.05	SB: Unclear risk
		*n* = 9;	20 g;		AB: Low risk for all outcomes
		age mean 10.8 years;	orally;		DB: Low risk for weight + High risk for all other outcomes
		3 females (33.3%), 6 males (66.7%);	daily;		CF: High risk
		not reported	28 days;		
			at the beginning and the end of the intervention after 28 days		
Poustie et al., 2006 (28)	Multicenter randomized controlled trial	Cystic fibrosis;	Protein energy supplements;	Weight *Δ* weight centile mean 0.83 (SD 10.96) supplement group vs. mean −1.00 (SD 7.14) standard care group, mean difference 1.83 (95% CI −1.79 to 5.45), *p* = 0.32 Height *Δ* height centile mean −0.53 (SD 6.94) supplement group vs. mean 1.18 (SD 5.62) standard care group, mean difference −0.65 (95% CI −3.12 to 1.83), *p* = 0.61 Mid-arm muscle circumference *Δ* mid-arm muscle circumference mean 0.76 (SD 1.37) supplement group vs. mean 0.62 (SD 1.00) standard care group, mean difference 0.14 (95% CI −0.34 to 0.61), *p* = 0.08 BMI *Δ* BMI centile mean 0.67 (SD 18.20) supplement group vs. mean −2.32 (SD 9.63) standard care group, mean difference 2.99 (95% CI −2.70 to 8.68), *p* = 0.30	SB: Low risk
		*n* = 102 (*n* = 50 in supplement group);	to increase energy intake by 20%;		AB: Low risk for all outcomes
		age range 5–12 years;	as drinks;		PB: High risk
		23 females (46.0%), 27 males (54.0%);	daily;		DB: Low risk for weight + High risk for all other outcomes
		not reported	12 months;		
			at baseline and after 12 months		
			routine dietetic advice and monitoring		
Shepherd et al., 1983 (29)	Prospective intervention study	Cystic fibrosis;	Protein and energy supplement;	Weight *Δ* standardized weight mean −1.23 (SEM 0.14) before vs. −0.70 (SEM 0.18) after supplementation, *p* < 0.05 Height *Δ* standardized height mean −0.91 (SEM 0.28) before vs. −0.53 (SEM 0.38) after supplementation, *p* < 0.02 Mid-upper arm circumference *Δ* mid-upper arm circumference (% reference for age and sex) mean 85 (SEM 2) before vs. 91 (SEM 2) after supplementation, *p* < 0.01 Triceps skinfold *Δ* triceps skinfold (% reference for age and sex) mean 52 (SEM 2) before vs. 63 (SEM 3) after supplementation, *p* < 0.005 Lean body mass *Δ* total body potassium (g) mean 59.8 (SEM 6.6) before vs. 70.4 (SEM 8.7) after supplementation, *p* < 0.01 *Δ* total body potassium as % reference body mass (x10−4) mean 1.68 (SEM 0.10) before vs. 1.95 (SEM 0.09) after supplementation, *p* < 0.01 Muscle mass *Δ* muscle mass (kg) mean 8.21 (SEM 0.61) before vs. 9.69 (SEM 1.27) after supplementation, *p* < 0.01 *Δ* muscle mass as % reference body mass mean 22.7 (SEM 2.3) before vs. 26.5 (SEM 2.3) after supplementation, *p* < 0.01 Body composition Improvements in weight and height (*p* < 0.05 and *p* < 0.01, respectively) attributed to increments in body fat [both as a percentage actual body mass (*p* < 0.05) and as a percentage reference body mass (*p* < 0.01)] and muscle mass (*p* < 0.01), including total body potassium as a percentage reference body mass (*p* < 0.01) Increase in total body potassium of mean 17.4% and increase in weight of mean 8.7%, with individual increases in total body potassium and muscle mass in all patients, indicating lean body mass accretion	SB: Unclear risk
		*n* = 7;	to increase protein and energy intake by 20–40%;		AB: High risk for muscle mass and lean body mass + Low risk for all other outcomes
		age range 5.2–13.2 years;	as drip-feed or drinks;		DB: Low risk for weight/ body mass, muscle mass and lean body mass + High risk for all other outcomes
		3 females (42.9%), 4 males (57.1%);	daily;		CF: High risk
		not reported	6 months;		
			before and after 6 months of supplementation		
Motil et al., 1982 (27)	Prospective intervention study	Crohn disease;	Protein and energy supplement;	Weight *Δ* weight (cm/month) mean 0.21 (SEM 0.09) [mean 10.0 (SEM 1.4) months] prior to vs. mean 1.22 (SEM 0.25) [mean 7.0 (SEM 0.8) months] after supplementation, *p* < 0.05 Height *Δ* height (cm/month) mean 0.10 (SEM 0.08) [mean 10.0 (SEM 1.4) months] prior to vs. mean 0.50 (SEM 0.16) [mean 7.0 (SEM 0.8) months] after supplementation, *p* < 0.05	SB: Unclear risk
		*n* = 6;	to increase protein and energy intake by 40%;		AB: High risk for all outcomes
		age mean 15.0 years;	via nasogastric tube or gastrostomy;		DB: Low risk for weight + High risk for height
		6 males (100.0%);	every night;		CF: High risk
		duration of Crohn disease from date of diagnosis ranged between 2 and 5 years	7 months;		
			at baseline and after 7 months for patients		
Eveleens et al., 2019 (25)	Retrospective database study	Children with a prolonged PICU stay;	Protein and energy-enriched formula;	Weight *Δ* body weight (g/kg/day) overall median increase 5.80 (IQR: 3.28–9.04) (and in patients between the age 0–3 months (*n* = 40) median increase 7.54 (IQR: 4.70–10.47), 3–6 months (*n* = 13) median increase 4.49 (IQR: 1.48–5.82) and 6–12 months (*n* = 17) median increase 3.88 (IQR: 2.92–6.18), significance level not reported Weight-for-age *Δ* WFA Z-score mean increase 0.48 (SD 1.10), *p* < 0.001 Number with WFA Z-score < −2 decreased from 33 (47%) to 23 (33%) Lower WFA Z-score at start associated with higher increase in WFA Z-score during PE-formula use [*r*^2^ = 0.26; *β* −0.35; 95% CI = −0.50 to −0.19; *p* < 0.001]. Other predictive baseline variables (e.g., WFA Z-score at birth, respiratory diagnosis, corrected age at start and reason to start) not associated with changes in WFA Z-score during PE-formula use	SB: Unclear risk
		*n* = 70;	to reach twice of individual calculated resting energy expenditure;		AB: Low risk for weight
		age range 37 post-menstrual weeks—12 months;	continuous and/or portion enteral feeding;		DB: Low risk for weight
		34 females (48.6%), 36 males (51.4%);	daily;		CF: High risk
		from as soon as possible, preferably the day after admission until weaning from ventilation or when the weight goal was achieved	median 29.2 days;		
			at the start and end of PE-formula use		

AB, attrition bias; BMI, body mass index; CF, confounding; CI, confidence interval; DB, detection bias; IQR, interquartile range; NS, not significant; PICU, pediatric intensive care unit; PB, performance bias; SB, selection bias; SD, standard deviation; SEM, standard error of mean; WFA, weight-for-age.

**Table 2:** Summary of evidence on physical fitness measurements (*n* = 3 studies).

Study (author; year)	Study design	Patients (disease; number; age; gender; follow-up duration)	Intervention [type of supplement; dose; way of supplementation; frequency; duration; timing; standard of care (for randomized controlled trials only)]	Main results	Risk of bias
Lands et al., 2010 (26)	Prospective intervention cohort study	Cystic fibrosis;	Pressurized whey;	Pulmonary function Δ FEV_1_ (% predicted) mean 81 pretreatment vs. mean 91 posttreatment, mean increase 10.7 (SD 13.77), *p* < 0.05 Δ RV/TLC (%) mean increase 0.4 (SD 6.2), NS	SB: Unclear risk
		*n* = 9;	20 g;		AB: Low risk for all outcomes
		age mean 10.8 years;	orally;		DB: Low risk for all outcomes
		3 females (33.3%), 6 males (66.7%);	daily;		CF: High risk
		not reported	28 days;		
			at the beginning and the end of the intervention after 28 days		
Poustie et al., 2006 (28)	Multicenter randomized controlled trial	Cystic fibrosis;	Protein energy supplements;	Pulmonary function Δ FEV_1_ (% predicted) mean −3.41 (SD: 13.50) supplement group vs. mean −1.50 (SD: 14.89) standard care group, mean difference −1.91 (95% CI −8.73 to 4.93), *p* = 0.58 Δ FVC (% predicted) mean 0.06 (SD: 17.82) supplement group vs. mean −5.21 (SD: 20.02) standard care group, mean difference 5.28 (95% CI −3.93 to 14.48), *p* = 0.26 Functional status Δ activity (% of day active) mean −4.97 (SD: 9.77) supplement group vs. mean −4.89 (SD: 10.70) standard care group, mean difference −0.07 (95% CI −4.1 to 3.96), *p* = 0.97	SB: Low risk
		*n* = 102 (*n* = 50 in supplement group);	to increase energy intake by 20%;		AB: Low risk for all outcomes
		age range 5–12 years;	as drinks;		PB: High risk
		23 females (46.0%), 27 males (54.0%);	daily;		DB: Low risk all outcomes
		not reported	12 months;		
			at baseline and after 12 months		
			routine dietetic advice and monitoring		
Shepherd et al., 1983 (29)	Prospective intervention study	Cystic fibrosis;	Protein and energy supplement;	Clinical outcomes Δ clinical scores mean 68 total (range 35–79), 48 pulmonary, 19 general before vs. 74 total, 51 pulmonary, 22 general after supplementation, *p* < 0.01, *p* < 0.05 and *p* < 0.001 respectively	SB: Unclear risk
		*n* = 7;	to increase protein and energy intake by 20–40%;		AB: Low risk for clinical scores
		age range 5.2–13.2 years;	as drip-feed or drinks;		DB: High risk for clinical scores
		3 females (42.9%), 4 males (57.1%);	daily;		CF: High risk
		not reported	6 months;		
			before and after 6 months of supplementation		

AB, attrition bias; CF, confounding; CI, confidence interval; DB, detection bias; FEV_1_, forced expiratory volume in one second; FVC, forced vital capacity; NS, not significant; PB, performance bias; RV, residual volume; SB, selection bias; SD, standard deviation; TLC, total lung capacity.

**Table 3:** Summary of evidence on intervention-related adverse effects (*n* = 3 studies).

Study (author; year)	Study design	Patients (disease; number; age; gender; follow-up duration)	Intervention [type of supplement; dose; way of supplementation; frequency; duration; timing; standard of care (for randomized controlled trials only)]	Main results	Risk of bias
Lands et al., 2010 (26)	Prospective intervention cohort study	Cystic fibrosis;	Pressurized whey;	Laboratory parameters Δ CRP (pg/mL) mean decrease 3.4 (SD 10.95), NS Δ total white blood cell count, percentage of neutrophils, absolute neutrophil count, whole blood glutathione measures, IL-6 and IL-8 response and hepatic and renal function parameters, NS	SB: Unclear risk
		*n* = 9;	20 g;		AB: High risk for whole blood glutathione measures + Low risk for all other outcomes
		age mean 10.8 years;	orally;		DB: Low risk for blood values
		3 females (33.3%), 6 males (66.7%);	daily;		CF: High risk
		not reported	28 days;		
			at the beginning and the end of the intervention after 28 days		
Poustie et al., 2006 (28)	Multicenter randomized controlled trial	Cystic fibrosis;	Protein energy supplements;	Gastrointestinal symptoms Δ gastrointestinal symptoms score mean −0.42 (SD 2.06) supplement group vs. mean −0.62 (SD: 2.03) standard care group, mean difference 0.20 (95% CI −0.61 to 1.00), *p* = 0.63	SB: Low risk
		*n* = 102 (*n* = 50 in supplement group);	to increase energy intake by 20%;		AB: Low risk for gastrointestinal symptoms score
		age range 5–12 years;	as drinks;		PB: High risk
		23 females (46.0%), 27 males (54.0%);	daily;		DB: High risk for gastrointestinal symptoms score
		not reported	12 months;		
			at baseline and after 12 months		
			routine dietetic advice and monitoring		
Shepherd et al., 1983 (29)	Prospective intervention study	Cystic fibrosis;	Protein and energy supplement;	Tolerance Reasonably well tolerance Compliance Good compliance Complications Occasional complications with drip-feeds, including vomiting	SB: Unclear risk
		*n* = 7;	to increase protein and energy intake by 20–40%;		AB: Low risk for all outcomes
		age range 5.2–13.2 years;	as drip-feed or drinks;		DB: High risk for all outcomes
		3 females (42.9%), 4 males (57.1%);	daily;		CF: High risk
		not reported	6 months;		
			before and after 6 months of supplementation		

AB, attrition bias; CF, confounding; CI, confidence interval; CRP, C-reactive protein; DB, detection bias; GRV, gastric residual volume; IL, interleukin; IQR, interquartile range; NS, not significant; PICU, pediatric intensive care unit; PB, performance bias; SB, selection bias; SD, standard deviation.

**Table 4:** Summary of effects of protein supplementation on anthropometric and body composition measurements, physical fitness measurements and intervention-related adverse effects.

Study (author; year)	Anthropometric and body composition measurements	Physical fitness measurements	Intervention-related adverse effects
Lands et al., 2010 (26) [pressurized whey; children with CF (*n* = 9)]			
Poustie et al., 2006* (28) [supplement to increase energy intake; children with CF (*n* = 50)]			
Shepherd et al., 1983 (29) [supplement to increase protein and energy intake; children with CF (*n* = 7)]			
Motil et al., 1982 (27) [supplement to increase protein and energy intake; children with CD (*n* = 6)]			
Eveleens et al., 2019 (25) [protein and energy-enriched formula; children with prolonged PICU stay (*n* = 70)]			

CD, Crohn disease; CF, cystic fibrosis; PICU, pediatric intensive care unit.

In this table, for the randomized controlled trial (*), the effect of the intervention in patients is reported vs. the effect of the intervention in controls while, for observational studies, the effect of the intervention before vs. after supplementation in patients is reported.

Dark green indicates a significant improvement all outcomes of the study, light green indicates a significant improvement in at least one of the outcomes, yellow indicates no significant change in the outcomes of the study, grey indicates an unknown significance level, no cell color indicates that the outcome was not assessed by the study.

### Effect on anthropometric and body composition measurements

All five studies reported an effect of the intervention on anthropometric and body composition measurements (25–29). The effect of daily supplementation of pressurized whey (20 g) for 28 days in nine children with CF was studied in a prospective intervention study (26). This study found a significant increase in BMI *z*-score (*p* < 0.05) but no significant changes in weight (*p* > 0.1), lean body mass and body fat percentage were reported.

The effect of protein and energy supplements to increase (protein and) energy intake ranging from 20 to 40% was examined by two studies in children with CF (28, 29) and by one study in children with CD (27). The way of supplementation varied between studies. In one of the studies in children with CF, fifty children received protein energy supplements to increase energy intake by 20% daily for twelve months. In this RCT, the changes in weight (*p* = 0.32) and height centile (*p* = 0.61), mid-arm muscle circumference (*p* = 0.08) and BMI (*p* = 0.30) over twelve months of daily supplementation were not significantly different compared with changes in children with CF in the control group (*n* = 52) who only received routine dietary advice and monitoring (28). The other study in children with CF (*n* = 7) who received a protein and energy supplement to increase protein and energy intake by 20%–40% daily for six months was a prospective intervention study (29). This study found significant improvements in standardized weight (*p* < 0.05) and height (*p* < 0.02), mid-upper arm circumference (*p* < 0.01), triceps skinfold (*p* < 0.005), total body potassium as a measure of lean body mass [both as absolute value (*p* < 0.01) and as percentage of reference body mass (*p* < 0.01)] and muscle mass [both as absolute value (*p* < 0.01) and as percentage of reference body mass (*p* < 0.01)] after six months of daily supplementation. The significant improvements in weight and height were attributed to increments in body fat [as percentage of actual (*p* < 0.05) as well as reference body mass (*p* < 0.01)] and muscle mass (*p* < 0.01) including total body potassium as a percentage of reference body mass (*p* < 0.01). Their total body potassium increased with a mean of 17.4% compared with a mean increase of 8.7% in weight, with individual increases in total body potassium and muscle mass in all children, indicating lean body mass accretion. The study performed in children with CD (*n* = 6) was a prospective intervention study which reported that weight (*p* < 0.05) and height (*p* < 0.05) gain respectively were nearly six and five times larger after seven months of supplementation compared with ten months prior to supplementation (27).

The effect of receiving a PE-formula daily to reach twice the individual calculated resting energy expenditure until weaning from ventilation or when the weight goal was achieved (median duration 29.2 days) was studied in 70 children with a prolonged PICU stay in a retrospective database study (25). This study found that the intervention resulted in a significant increase in weight-for-age (WFA) Z-score (*p* < 0.001) and a median increase in body weight. The number of children with WFA Z-score <−2 decreased from 33 (47%) at the start to 23 (33%) at the end of PE-formula use. A lower WFA Z-score at the start was associated with a significant higher increase in WFA Z-score during PE-formula use (*p* < 0.001).

### Effect on physical fitness measurements

Three studies determined the effect of the intervention on physical fitness measurements (26, 28, 29). One study determined the effect of pressurized whey (20 g) supplementation daily for 28 days in nine children with CF (26). This study had a prospective intervention design and found a significant increase in forced expiratory volume in one second (FEV_1_) (i.e., airflow limitation) (*p* < 0.05) whereas no significant change in residual volume (RV)/total lung capacity (TLC) (i.e., air-trapping) was reported.

In the other two studies, the effect of protein and energy supplements to increase (protein and) energy intake by 20%–40% was examined in children with CF (28, 29). The way of supplementation varied between the studies. One of these studies, in which the children with CF (*n* = 50) received protein energy supplements to increase energy intake by 20% daily for twelve months, was a RCT (28). Changes over twelve months in FEV_1_ (*p* = 0.58) and forced vital capacity (FVC) (*p* = 0.26) as percentages of predicted values were not significantly different from changes in children with CF (*n* = 52) in the control group who only received routine dietary advice and monitoring. This study also found that the change in activity (*p* = 0.97) (as a percentage of the day being active) was not significantly different from the change in children in the control group. The other study, in which seven children with CF received a protein and energy supplement to increase protein and energy intake by 20%–40% daily for six months, was a prospective intervention study (29). This study reported that average clinical scores significantly improved [total score (*p* < 0.01), pulmonary (*p* < 0.05), general (*p* < 0.001)] in all but one case.

### Effect on intervention-related adverse effects

Three studies reported on possible adverse effects associated with the intervention (26, 28, 29). Daily supplementation of pressurized whey (20 g) for 28 days in nine children with CF in a prospective intervention study did not result in significant changes in C-reactive protein (CRP) nor in total white blood cell count, percentage of neutrophils, absolute neutrophil count, whole blood glutathione measures, IL-6 and IL-8 response and hepatic or renal function parameters (26).

The effect of protein and energy supplements to increase (protein and) energy intake by 20%–40% was reported in two studies in children with CF with varying ways of supplementation (28, 29). The study that examined the effect of protein energy supplements to increase energy intake by 20% daily for twelve months in children with CF (*n* = 102) was a RCT (28). This study reported no significant change in gastrointestinal symptoms in the intervention group (*n* = 50) compared to children with CF (*n* = 52) in the control group who only received routine dietary advice and monitoring (28). The other study in children with CF (*n* = 7) who received a protein and energy supplement to increase protein and energy intake by 20%–40% for six months was a prospective intervention study (29). All children in this study tolerated the supplements reasonably well and compliance was good. Occasional complications were encountered but these were not persistent.

### Risk of bias of included studies and GRADE quality of evidence

Risk of bias varied in the included studies but none of the studies were free of any bias. A detailed description of the risk of bias assessment for all included studies is shown in Supplementary material 4.

Regarding the GRADE assessment, pooling of the results was not valid because only one study was available for each specific outcome in relation to study design, patients and intervention. Therefore, inconsistency in terms of differences across the study results was not applicable. Publication bias was unlikely as we performed an extensive search and identified studies showing favorable effects as well as studies that reported no effects of the intervention on the outcomes. However, for each outcome, we downgraded 1 level for indirectness as no studies in children with cancer were available. Moreover, we downgraded 2 levels for imprecision as for each specific outcome there was only one study available with a certain study design, patient group and intervention and all but one of these studies included only a relatively small number of patients. For study limitations the exact number of downgrading differed between outcomes but was at least minus 1 for the RCT and minus 2 for the observational studies. There were no reasons for upgrading any of the outcomes. Therefore, for all outcomes, the level of evidence was of very low quality.

## Discussion

In this systematic review, we did not identify any eligible study examining the potential effect of protein supplementation on anthropometrics, body composition and physical fitness in children with cancer. However, we identified a limited number of studies (*n* = 5) examining its potential in other children with chronic health conditions or critical illness. The modest amount of evidence included in this review tentatively suggests that protein supplementation might be a valuable addition to medical practice in improving anthropometrics, body composition and physical fitness. Chronically and critically ill children have in common with children with cancer that their disease often requires long-term treatment with catabolic effects, possibly including chemotherapy and/or radiotherapy, might involve prolonged hospitalizations and might lead to side effects such as immobilization, pain, nausea, reduced appetite, changes in smell and taste, altered (protein) metabolism and a suboptimal nutritional status. Moreover, heterogeneity in physical complaints exists between children with cancer. Therefore, we expect comparable changes in anthropometrics, body composition and physical fitness during treatment of the diseases in these populations. Subsequently, we anticipate learning from existing evidence in chronically and critically ill children and to apply this knowledge in children with cancer. Nonetheless, as the evidence identified in this systematic review was of very low quality and results are thus uncertain, more high-quality research is needed, especially in children with cancer.

All five included studies, assessing different protein interventions, reported on anthropometric and body composition measurements (25–29). The RCT showed no significant changes in any of the measurements (28), while results in the observational studies showed varying results, depending on the measurements that were assessed. In all four observational studies, a significant improvement in at least one of the measurements was reported (25–27, 29). Nonetheless, not all measurements assessed in the studies showed an effect. In adult cancer patients, some preliminary findings pointing towards favorable effects of protein supplementation on anthropometrics and body composition were reported previously (16, 30, 31).

Three studies assessed the effect of different protein interventions on physical fitness measurements (26, 28, 29). The RCT did not find significant changes in physical fitness measurements (28). In contrast, the two observational studies reported a significant improvement in at least one of the physical fitness measurements assessed (26, 29). All three studies assessing physical fitness measurements were conducted in children with CF, who might have worse lung function compared to children with cancer and in whom reduced lung function is the primary cause of early death (28). As these children might have lower baseline values and therefore possibly have a larger potential to benefit from protein supplementation, it is of concern to investigate whether the beneficial effects of protein supplementation on physical fitness also translate to children with cancer.

Three studies, examining different protein interventions, reported on adverse effects of the intervention (26, 28, 29). These studies did not report substantial adverse effects apart from occasional complications with drip-feeds. Therefore, the results of the current review regarding intervention-related adverse effects are encouraging, although, especially when modifying and assessing the effect of the intervention, possible intervention-related adverse effects should still be monitored and deserve further investigation, in particular in children with cancer. Adverse effects that are important to monitor include laboratory parameters (e.g., blood cell count and organ function parameters), immune function, wound healing, drug metabolism, chemotherapeutic tolerance and/or overall survival.

The evidence included in this review cautiously indicates that protein supplementation may have a place in improving anthropometrics, body composition and physical fitness in children with cancer. Furthermore, previous literature showed that critically ill children receiving a higher percentage of prescribed energy and protein dose had improved survival outcomes (32) and in adult cancer patients, low physical activity and diminished body composition were associated with poor outcomes and survival (31, 33, 34). Therefore, protein supplementation might also contribute to enhanced survival.

Even though this review provides insights into the existing evidence on the potential of protein supplementation to improve anthropometrics, body composition and physical fitness, several limitations of the available evidence should be taken into consideration. First, no study examining the effect of protein supplementation on anthropometrics, body composition and physical fitness in children with cancer was identified. Despite previously mentioned similarities between children with cancer and other chronically and critically ill children, differences between these populations exist. For example, children with CF and CD might have a worse protein absorption and require a higher protein dose. In addition, children with CD and those admitted to the PICU might be in an even higher catabolic state due to inflammation and acute stress respectively compared to children with cancer. Therefore, generalizability of the results of the current review to children with cancer is limited. Second, each of the included studies was rated as having high risk of bias in at least one domain and the evidence was of very low quality for all assessed outcomes. As a result, caution is warranted when interpreting the evidence. Moreover, only a limited number of studies in chronically (*n* = 4) and critically ill children (*n* = 1) could be included. Furthermore, in three of them the number of patients receiving a protein intervention was very small (*n* ≤ 10) (26, 27, 29). Additionally, only one of the studies was a RCT (28). The other studies were observational studies assessing the effect of protein supplementation before and after supplementation. Such studies might give an indication of the effect of the intervention but determining whether changes originate from the intervention or from other factors might remain difficult (35). Therefore, results should always be interpreted with caution. In addition, heterogeneity existed between the included studies; the type of disease differed (i.e., CF, CD and prolonged PICU stay due to varying diagnoses) and the age range in the study by Eveleens et al. (25) (i.e., 37 post-menstrual weeks—twelve months) was clearly lower compared to the other studies. Furthermore, the protein interventions were diverse in terms of type of supplement, dose, way of supplementation, frequency and duration.

The effect of protein supplementation deserves future investigation. Of primary interest would be to examine whether the evidence included in this review translates to children with cancer by first assessing the feasibility of protein supplementation and, subsequently, its effectiveness through a RCT. When doing so, it is also important to rule out potential adverse effects. If protein supplementation is found to be feasible in children with cancer and the preferred way of supplementation is established, future studies should focus on determining the optimal dose, frequency and duration as these factors might influence the extent of its potential benefits and possible adverse effects. Preferably, the supplement should contain only proteins as an adequate protein intake is needed to support physiological functions in line with children's sex, age and disease. On the other hand, the influence of an increased energy intake on anthropometrics, body composition and physical fitness should be minimized. While some children are at increased risk for underweight, others are at risk for developing overweight during childhood cancer and its treatment (9). Therefore, careful consideration is required when prescribing supplements. One possible approach could be to reduce dietary fat intake while increasing protein intake to keep the total energy content of a child's diet similar. However, a balanced diet that meets the requirements of all macronutrients is necessary for anabolic and regenerative functions. Ideally, and when feasible, increasing physical activity levels could help compensate for the higher energy intake. This might not only benefit anthropometrics, body composition and physical fitness but also enhance the potential synergistic effect of combining protein supplementation with exercise interventions. As the optimal protein requirement as well as the protein intake of children with cancer remains unknown, assessing this in future research would be valuable. Nonetheless, increasing protein dose might not only cause beneficial effects but might also cause harm. For example, consumption of a low carbohydrate high dietary protein diet was recently shown to be associated with a shorter health- and lifespan in a mouse model for accelerated aging and hyperresponsive to the anti-aging effect of dietary restriction (36). However, the dose that was used in this recent study was much higher than will be ingested by children with cancer. Lastly, a common side effect of childhood cancer includes impaired renal function (37), which might be aggravated by excess protein intake.

## Conclusion

Currently, evidence on the potential of protein supplementation on anthropometrics, body composition and physical fitness is scarce and not available in children with cancer. However, taken together, the limited evidence included in this review tentatively suggests that protein supplementation might be a valuable addition to medical practice in improving anthropometrics, body composition and physical fitness. Future high-quality studies are needed to determine its feasibility and effectiveness in children with cancer.

## Data Availability

The original contributions presented in the study are included in the article/Supplementary Material, further inquiries can be directed to the corresponding author.
