# Supplementary material for: Protein supplementation in pediatric oncology: a systematic review revealing a lack of evidence and insights from other chronically and critically ill children
Source: Front Pediatr. 2025 Dec 10;13:1724658. doi: 10.3389/fped.2025.1724658 (PMC12728054; doi:10.3389/fped.2025.1724658)
Supplement: Supplementary file 1 [file Supplementaryfile1.docx]

*Supplementary material 1. Search strategy PubMed/MEDLINE for protein supplementation in children with cancer (from 1945 to 14 May 2025).*

1. **Childhood cancer:**

leukemia OR leukemi* OR leukaemi* OR “childhood ALL” OR AML OR (leukemia, lymphocytic, acute[mh]) OR (leukemia, lymphocytic, acute*) OR lymphoma OR lymphom* OR hodgkin OR hodgkin* OR T-cell OR B-cell OR non-hodgkin OR non-hodgkin* OR sarcoma OR sarcom* OR sarcoma, Ewing's OR Ewing* OR osteosarcoma OR osteosarcom* OR wilms tumor OR wilms* OR nephroblastom* OR neuroblastoma OR neuroblastom* OR rhabdomyosarcoma OR rhabdomyosarcom* OR teratoma OR teratom* OR hepatoma OR hepatom* OR hepatoblastoma OR hepatoblastom* OR PNET OR medulloblastoma OR medulloblastom* OR PNET* OR neuroectodermal tumors, primitive OR retinoblastoma OR retinoblastom* OR meningioma OR meningiom* OR glioma OR gliom* OR brain tumor OR brain tumor* OR brain tumour* OR brain cancer* OR brain neoplasm* OR intracranial neoplasm* OR brain neoplasms OR central nervous system neoplasm OR central nervous system neoplasms OR central nervous system neoplasm* OR central nervous system tumor OR central nervous system tumour OR central nervous system tumor* OR central nervous system tumour* OR pediatric oncology OR paediatric oncology OR childhood cancer OR childhood tumor OR childhood tumors

1. **Children^1^:**

infan* OR newborn* OR new-born* OR perinat* OR neonat* OR baby OR baby* OR babies OR toddler* OR minors OR minors* OR boy OR boys OR boyfriend OR boyhood OR girl* OR kid OR kids OR child OR child* OR children* OR schoolchild* OR schoolchild OR school child[tiab] OR school child*[tiab] OR adolescen* OR juvenil* OR youth* OR teen* OR under*age* OR pubescen* OR pediatrics[mh] OR pediatric* OR paediatric* OR peadiatric* OR school [tiab] OR school*[tiab] OR prematur* OR preterm*

1. **Protein supplementation:**

Dietary proteins [mh] OR dietary protein [tiab] OR dietary proteins [tiab] OR protein supplementation [tiab] OR protein supplements [tiab] OR protein supplement [tiab] OR protein supplement* [tiab]

**Combination: 1 AND 2 AND 3**

*Supplementary material 2. Search strategy PubMed/MEDLINE for protein supplementation in chronically and critically ill children (from 1945 to 14 May 2025).*

1. **Children^1^:**

infan* OR newborn* OR new-born* OR perinat* OR neonat* OR baby OR baby* OR babies OR toddler* OR minors OR minors* OR boy OR boys OR boyfriend OR boyhood OR girl* OR kid OR kids OR child OR child* OR children* OR schoolchild* OR schoolchild OR school child[tiab] OR school child*[tiab] OR adolescen* OR juvenil* OR youth* OR teen* OR under*age* OR pubescen* OR pediatrics[mh] OR pediatric* OR paediatric* OR peadiatric* OR school [tiab] OR school*[tiab] OR prematur* OR preterm*

1. **Chronically and critically illness:**

critical illness OR critical illnesses OR critical illness* OR critically ill OR critical care OR intensive care OR intensive care unit OR intensive care units OR intensive care unit* OR ICU OR ICUs OR NICU OR NICUs OR PICU OR PICUs OR intensive care, neonatal OR neonatal intensive care OR pediatric intensive care OR paediatric intensive care OR intensive Care Units, Pediatric OR pediatric intensive care unit OR pediatric intensive care units OR pediatric intensive care unit* OR pediatric ICU OR pediatric ICUs OR paediatric intensive care unit OR paediatric intensive care units OR paediatric intensive care unit* OR paediatric ICU OR paediatric ICUs OR intensive care units, neonatal OR neonatal intensive care unit OR neonatal intensive care units OR neonatal intensive care unit* OR neonatal ICU OR neonatal ICUs OR newborn intensive care unit OR newborn intensive care units OR newborn intensive care unit* OR newborn ICU OR newborn ICUs OR cystic fibrosis OR cystic fibro* OR CF OR mucoviscidosis OR mucoviscido* OR fibrocystic disease or fibrocystic diseases OR fibrocystic diseas* OR bronchopulmonary dysplasia OR bronchopulmonary dysplas* OR BPD OR cerebral palsy OR cerebral palsies OR cerebral pals* OR CP OR “Little Disease” OR “Little's Disease” OR spastic diplegia OR spastic diplegias OR spastic dipleg* OR acquired brain injury OR acquired brain injuries OR acquired brain injur* OR ABI OR Inflammatory Bowel Diseases OR Inflammatory Bowel Disease OR Inflammatory Bowel Diseas* OR IBD OR Colitis, Ulcerative OR Ulcerative Colitis OR ulcerative colit* OR Colitis Gravis OR colitis grav* OR Idiopathic Proctocolitis OR Idiopathic Proctocolit* OR Crohn Disease OR Crohn's Disease OR Crohns Disease OR Crohn Diseas* OR Crohn's Diseas* OR Crohns Diseas* OR Crohn's Enteritis OR crohn’s enterit* OR ileocolitis OR ileocolit* OR ileitis OR ileitides OR ileit* OR Granulomatous Enteritis OR Granulomatous Enterit* OR Granulomatous Colitis OR Granulomatous Colit* OR heart diseases OR heart disease OR heart diseas* OR cardiac diseases OR cardiac disease OR cardiac diseas* OR cardiac disorder OR cardiac disorders OR cardiac disord* OR heart disorder OR heart disorders OR heart disord* OR cardiomyopathies OR cardiomyopathy OR cardiomyopath* OR myocardial disease OR myocardial diseases OR myocardial diseas* OR myocardiopathies OR myocardiopathy OR myocardiopath* OR heart defects, congenital OR congenital heart defect OR congenital heart defects OR congenital heart defect* OR heart abnormality OR heart abnormalities OR heart abnormal* OR malformation of heart

1. **Protein supplementation:**

Dietary proteins [mh] OR dietary protein [tiab] OR dietary proteins [tiab] OR protein supplementation [tiab] OR protein supplements [tiab] OR protein supplement [tiab] OR protein supplement* [tiab]

**Combination: 1 AND 2 AND 3**

*Supplementary material 3. Risk of bias criteria for (randomized) controlled trails and observational studies.*

Randomized controlled and controlled clinical trials*^2^*

Each bias item should be scored as low risk, high risk or unclear risk (no overall scores should be calculated); attrition bias and detection bias should be scored for each outcome separately.

|  | **Internal validity** |
| --- | --- |
| **Study group** | **Selection bias (Is the study group representative?):**  *Low risk/ high risk/ unclear risk*  Low risk if  there was random sequence allocation (the investigators describe an adequate random component in the sequence generation process) and allocation concealment (patients and investigators enrolling patients could not foresee assignment). |
| **Follow-up** | **Attrition bias (Is the follow-up adequate?):**  *Low risk/ high risk/ unclear risk*  Low risk if  the outcome was assessed for more than 90% of the study group in each treatment arm. |
| **Outcome** | **Performance bias (Are the patients and personnel blinded from knowledge of which intervention was received?):**  *Low risk/ high risk/ unclear risk*  Low risk if  the patients and personnel were blinded from knowledge of which intervention was received (no placebo is considered not blinded). |
|  | **Detection bias (Are the outcome assessors blinded from knowledge of which intervention was received?):**  *Low risk/ high risk/ unclear risk*  Low risk if  the outcome assessors were blinded from knowledge of which intervention was received (blinded if outcome measurement unlikely to be influenced by lack of blinding: laboratory tests). |

Observational studies*

Each bias item should be scored as low risk, high risk or unclear risk (no overall scores should be calculated); attrition bias and detection bias should be scored for each outcome separately.

********Based on previously described checklists according to evidence-based medicine criteria^3,4^.*

|  | **Internal validity** |
| --- | --- |
| **Study group** | **Selection bias (Is the study group representative?):**  *Low risk/ high risk/ unclear risk*  Low risk if  the study group consisted of more than 90% of the original cohort of eligible patients  or it was a random sample with respect to treatment and important prognostic factors (i.e. body composition at start of intervention, gender, age, follow-up). |
| **Follow-up** | **Attrition bias (Is the follow-up adequate?):**  *Low risk/ high risk/ unclear risk*  Low risk if  the outcome was assessed for more than 90% of the study group. |
| **Outcome** | **Detection bias (Are the outcome assessors blinded for important determinants related to the outcome?):**  *Low risk/ high risk/ unclear risk*  Low risk if  the outcome assessors were blinded for important determinants related to the outcome. |
| **Risk estimation** | **Confounding (Are the analyses adjusted for important confounders?):**  *Low risk/ high risk/ unclear risk*  Low risk if  important prognostic factors (i.e. body composition at start of intervention, gender, age, follow-up) were taken adequately into account. |

*Supplementary material 4. Evidence tables per included study with details on study characteristics, patients, intervention, main outcomes and additional remarks.*

| **To study short-term effects of pressurized whey protein supplementation on nutritional status in children with cystic fibrosis (CF).** | | | | |
| --- | --- | --- | --- | --- |
| Lands et al. Dietary supplementation with pressurized whey in patients with cystic fibrosis. Journal of medicinal food 2010;13(1):77-82. | | | | |
| **Study characteristics** | **Patients** | **Intervention** | **Main outcomes** | **Additional remarks**  **Risk of bias assessment** |
| Study design:  Prospective intervention cohort study. | Number and type of patients:  n=9 children with CF, able to perform pulmonary function tests, have a baseline forced expiratory volume in 1 second (FEV_1_) >50% predicted and with no change in clinical status for the month preceding the study.  Exclusion criteria:  Patients with significant hepatic or renal impairment as judged by their treating physician, already taking a protein supplement, and with a documented allergy to cow's milk protein.  Type of disease:  Cystic fibrosis.  Treatment details:  Patients were instructed to take pancreatic enzyme supplements equivalent to what they take for a snack and asked to otherwise continue their habitual dietary habits.  Follow-up duration:  Not reported.  Age at testing intervention group:  Above the age of 6 years.  Mean 10.8 (SD 3.8) years.  Gender:  3 females (33.3%), 6 males (66.7%).  Controls:  No. | Type of supplement:  Pressurized whey.  Dose:  20 g (5 g/50 mL of applesauce).  Way of supplementation:  Oral in applesauce, taken apart from a meal.  Frequency:  Daily.  Duration:  28 days.  Standard of care:  Not applicable. | Anthropometric and body composition outcomes and assessment:   - Height measured on a stadiometer. - Weight measured on an electronic balance. - BMI was calculated (method not specified). - Lean body mass assessed by four-site (i.e. biceps, triceps, subscapular and supra-iliac) skinfold measurement. - Body fat percentage (method not specified).   Timing:  At the beginning and the end of the intervention after 28 days.  Anthropometric and body composition results:   - Height (cm) at baseline mean 141.0 (SD 24.69). - Weight (kg) at baseline mean 34.4 (SD 15.16). - Lean body mass (kg) at baseline mean 28.3 (SD 12.22). - Body fat percentage (%) at baseline mean 17.3 (SD 2.72). - BMI Z-score at baseline mean -0.69 (SD 0.698). - Change in weight (g) from pretreatment to posttreatment mean 480 (SD 832), p>0.1. - Change in lean body mass (g) from pretreatment to posttreatment mean 322 (SD 736), not significant. - Change in body fat percentage (%) from pretreatment to posttreatment mean 0.5 (SD 2.25), not significant. - Change in BMI Z-score from pretreatment (mean -0.69) to posttreatment (mean -0.52) mean 0.16 (SD 0.206), p<0.05.   Physical fitness outcomes and assessment:   - Lung function, including airflow limitation and air-tapping, assessed by spirometry and whole body plethysmography.   Timing:  At the beginning and the end of the intervention after 28 days.  Physical fitness results:   - Airflow limitation (FEV_1_) (% predicted) at baseline mean 80.1 (SD 16.78). - Air-trapping (RV/TLC) (%) at baseline mean 30.7 (SD 7.91), evident compared to normal (<25%). - Change in FEV_1_ (% predicted) from pretreatment (81) to posttreatment (91) mean 10.7 (SD 13.77), p<0.05. - Change in RV/TLC (%) from pretreatment to posttreatment mean 0.4 (SD 6.2), not significant.   Adverse effects outcomes and assessment:   - Total and differential white blood cell count, high-sensitivity CRP serum concentration, whole blood glutathione concentration, cytokine production and hepatic and renal function assessed by a blood draw.   Timing:  At the beginning and the end of the intervention after 28 days.  Adverse effects results:   - CRP (pg/mL) at baseline mean 0.7 (SD 0.74). - White blood cell count (10^9^/L) at baseline mean 8.65 (SD 3.48). - Relative neutrophil count (%) at baseline mean 50.8 (SD 16.48). - Change in CRP (pg/mL) from pretreatment to posttreatment mean 3.4 (SD 10.95), not significant. - 10 of the 13 patients (of which two children) with initial CRP values > 1.0 pg/mL showed decreased values at follow up, p<0.05. - Total white blood cell count, percentage of neutrophils, absolute neutrophil count, whole blood glutathione measures, IL-6 or IL-8 response and hepatic or renal function parameters did not change significantly. | Additional remarks:  Funding from the BREATHE Initiative of the Canadian Cystic Fibrosis Foundation.  Selection bias:  Unclear risk  Reason: Not reported if all eligible patients were included or it was a random sample.  Attrition bias:  High risk for whole blood glutathione measures + Low risk for all other outcomes  Reason: Whole blood glutathione measures were available in 6/9 (66.7%) of patients, all other outcomes were assessed in all patients.  Detection bias:  Low risk for weight and blood values + High risk for all other outcomes  Reason: Outcome assessors were not blinded but weight and blood values are objective outcomes.  Confounding:  High risk  Reason: Important prognostic factors were not taken into account in the analyses. |

Abbreviations: BMI = body mass index, CF = cystic fibrosis, CRP = C-reactive protein, FEV_1_ = forced expiratory volume in 1 second, IL = interleukin, RV = residual volume, SD = standard deviation, TLC = total lung capacity.

| **To investigate whether oral protein energy supplements in addition to dietary advice and monitoring, improve or prevent deterioration in the body mass index centile and other measures of spirometric lung function and activity levels of children with cystic fibrosis (CF) compared with dietary management alone.** | | | | |
| --- | --- | --- | --- | --- |
| Poustie et al. Oral protein energy supplements for children with cystic fibrosis: CALICO multicentre randomised controlled trial. BMJ (Clinical research ed.) 2006;322(7542):632-6. | | | | |
| **Study characteristics** | **Patients** | **Intervention** | **Main outcomes** | **Additional remarks**  **Risk of bias assessment** |
| Study design:  Multicenter randomized controlled trial.  Randomization method:  Random number tables and sequentially numbered, opaque envelopes were used to generate the randomisation code and for treatment group allocation respectively. Randomisation was stratified within each centre. | Number and type of patients:  n=102 moderately malnourished children with CF (i.e. body mass index of less than the 25th centile and more than the 0.4th centile, no increase in weight over the previous three months, a 5% decrease in weight from baseline over a period of less than six months), of which n=50 in the supplement group and n=52 in the standard care group.  Exclusion criteria:  Children who had cystic fibrosis related diabetes or liver disease or had a forced expiratory volume in one second of less than 30% of predicted for height and age or if, during the previous three months, they had been diagnosed as having cystic fibrosis or had received enteral nutrition.  Type of disease:  Cystic fibrosis.  Treatment details:  Not reported.  Follow-up duration:  Not reported.  Age at testing intervention group:  Mean 8.75 (SD 3.72) years. Range 5-12 years.  Gender:  23 females (46.0%), 27 males (54.0%).  Controls:  Age at testing mean 8.79 (SD 3.67) years. Range 5-12 years.  25 females (48.1%), 27 males (51.9%). | Type of supplement:  Eleven different oral protein energy supplements (i.e. Calshake, Clinutren, Complan, Enlive, Fortifresh, Fortjuice, Fortini, Fortisip, Fresubin, Resource, Scandishake). Children selected the supplements they liked. Supplements in addition to dietary advice and monitoring.  Dose:  Recommended amount to increase usual energy intake by 20%.  Way of supplementation:  Oral, taken as drinks.  Frequency:  Daily.  Duration:  12 months.  Standard of care:  Routine dietetic advice and monitoring. | Anthropometric and body composition outcomes and assessment:   - Change in BMI centile (method not specified). - Weight measured by portable scales. - Height measured by a stadiometer. - Mid-arm muscle circumference calculated using tricep skinfold thickness assessed with skinfold callipers and mid-upper arm circumference.   Timing:  At baseline and after 12 months.  Anthropometric and body composition results:   - BMI centile at baseline mean 34.27 (SD 23.96) for supplement group and mean 31.52 (SD 25.36) for standard care group. - Weight centile at baseline mean 25.07 (SD 20.37) for supplement group and mean 24.69 (SD 22.79) for standard care group. - Height centile at baseline mean 26.69 (SD 24.83) for supplement group and mean 28.15 (SD 26.93) for standard care group. - Change in BMI centile over 12 months mean 0.67 (SD 18.20) for supplement group and mean -2.32 (SD 9.63) for standard care group. No significant difference between the groups in mean change from baseline to 12 months mean difference 2.99 (95% CI -2.70 to 8.68), p=0.30. - Change in weight centile over 12 months mean 0.83 (SD 10.96) for supplement group and mean -1.00 (SD 7.14) for standard care group. No significant difference between the groups in mean change from baseline to 12 months mean difference 1.83 (95% CI -1.79 to 5.45), p=0.32. - Change in height centile over 12 months mean -0.53 (SD 6.94) for supplement group and mean 1.18 (SD 5.62) for standard care group. No significant difference between the groups in mean change from baseline to 12 months mean difference -0.65 (95% CI -3.12 to 1.83), p=0.61. - Change in mid-arm muscle circumference over 12 months mean 0.76 (SD 1.37) for supplement group and mean 0.62 (SD 1.00) for standard care group. No significant difference between the groups in mean change from baseline to 12 months mean difference 0.14 (95% CI -0.34 to 0.61), p=0.08.   Physical fitness outcomes and assessment:   - Spirometric lung function parameters (forced expiratory volume in one second (FEV) and forced vital capacity (FVC)) measured with a portable spirometer (only performed in children aged 5 and above (n=72)). - Activity levels assessed by the habitual activity estimation scale and expressed as percentage of 24 hours spent being active.   Timing:  At baseline and after 12 months.  Physical fitness results:   - FEV_1_ (% predicted) at baseline mean 81.34 (SD 16.16) for supplement group and mean 73.67 (SD 29.59) for standard care group. - Change in FEV_1_ (% predicted) over 12 months mean -3.41 (SD 13.50) for supplement group and mean -1.50 (SD 14.89) for standard care group. No significant difference between the groups in mean change from baseline to 12 months mean difference -1.91 (95% CI -8.73 to 4.93), p=0.58. - Change in FVC (% predicted) over 12 months mean 0.06 (SD 17.82) for supplement group and mean -5.21 (SD 20.02) for standard care group. No significant difference between the groups in mean change from baseline to 12 months mean difference 5.28 (95% CI -3.93 to 14.48), p=0.26. - Change in activity (% of day active) over 12 months mean -4.97 (SD 9.77) for supplement group and mean -4.89 (SD 10.70) for standard care group. No significant difference between the groups in mean change from baseline to 12 months mean difference -0.07 (95% CI -4.1 to 3.96), p=0.97.   Adverse effects outcomes and assessment:   - Gastrointestinal symptoms monitored with a questionnaire adapted from a validated tool.   Timing:  At baseline and after 12 months.  Adverse effects results:   - Change in gastrointestinal symptoms score over 12 months mean -0.42 (SD 2.06) for supplement group and mean -0.62 (SD 2.03) for standard care group. No significant difference between the groups in mean change from baseline to 12 months mean difference 0.20 (95% CI -0.61 to 1.00), p=0.63. | Additional remarks:  Funded by a grant from the UK Cystic Fibrosis Trust.  Selection bias:  Low risk  Reason: Adequate methods used for random sequence allocation and allocation concealment.  Attrition bias:  Low risk for all outcomes  Spirometry outcomes were assessed in 70/72 (97.2%) of patients aged 5 and above, all other outcomes were assessed in all patients.  Performance bias:  High risk  Reason: Patients were not blinded as no satisfactory placebo was available and personnel was not masked to allocation group.  Detection bias:  Low risk for weight + High risk for all other outcomes  Reason: Outcome assessors were not blinded but weight is an objective outcome. |

Abbreviations: BMI = body mass index, CF = cystic fibrosis, CI = confidence interval, FEV_1_ = forced expiratory volume in 1 second, FVC = forced vital capacity, SD = standard deviation.

| **To investigate changes in body composition following long-term nutritional supplementation with a peptide formula diet in growth-retarded children with cystic fibrosis (CF).** | | | | |
| --- | --- | --- | --- | --- |
| Shepherd et al. Changes in body composition and muscle protein degradation during nutritional supplementation in nutritionally growth-retarded children with cystic fibrosis. Journal of pediatric gastroenterology and nutrition 1983;2(3):439-46. | | | | |
| **Study characteristics** | **Patients** | **Intervention** | **Main outcomes** | **Additional remarks**  **Risk of bias assessment** |
| Study design:  Prospective intervention study. | Number and type of patients:  n=7 poorly nourished and growth-retarded (>1 SD below weight for age) children with CF despite conventional management. All had chronic pulmonary disease, but none had clinical or biochemical evidence of liver disease. During each study visit, they were in stable clinical condition and free of active pulmonary infection.  Exclusion criteria:  Not reported.  Type of disease:  Cystic fibrosis.  Treatment details:  Managed at home on conventional therapy (regular bronchodilators and physiotherapy, pancreatic supplements, and an encouraged high-energy, high-protein diet with vitamin supplements). Pancreatic replacement therapy was not altered.  Follow-up duration:  Not reported.  Age at testing intervention group:  Range 5.2-13.2 years.  Gender:  3 females (42.9%), 4 males (57.1%).  Controls:  n=8 healthy, height-matched controls.  Age range 5.5-13.4 years.  3 females (37.5%), 5 males (62.5%). | Type of supplement:  Dietary supplement using a balanced peptide formula (Vipep®, Tuta Laboratories, Lane Cove, Australia).  Dose:  The supplement increased protein and energy intakes by 20-40%. The formula contained 10% of total calories as protein (peptide enzymatic digest of fish protein), 22% as fat (including 2.5% linoleic acid in a 4:1 blend of medium-chain triglycerides and corn oil), 68% as carbohydrate (corn syrup solids), plus vitamins and minerals. The osmolality was 520 mOsmol/kg H_2_O and the renal solute load was 202 mOsmol/L at a caloric density of 1 kcal/ml. The total volume administered to each patient per day was 1,000-1,200 ml (1,000-1,200 kcal).  Way of supplementation:  At home supplementation. In three patients as a nocturnal intragastric drip-feed via 2 mm o.d. weighted silicone nasogastric tube. Four patients took flavored Vipep with additional pancreatic supplements as several drinks per day.  Frequency:  Daily.  Duration:  6 months.  Standard of care:  Not applicable. | Anthropometric and body composition outcomes and assessment:   - Anthropometric measurements, including weight, height and mid-upper arm circumference, were obtained (method not specified). - Tricep skinfold thickness measurements taken with calipers. - Body composition assessed by estimating body mass, body fat, fat-free mass, muscle mass and total body potassium. Body fat and fat-free mass derived from four (i.e triceps, biceps, subscapular, suprailiac) skinfold measurements. Muscle mass calculated from urinary creatinine excretion. Lean body mass assessed from potassium content. Total body potassium estimated from measurement of potassium.   Timing:  Before and after 6 months of supplementation.  Anthropometric and body composition results:   - Standardized weight at baseline mean -1.23 (SEM 0.14) for patients and mean 0 (SEM 0.09) for controls, p<0.001. - Standardized height at baseline mean -0.91 (SEM 0.28) for patients and mean 0.19 (SEM 0.09) for controls, p<0.01. - Mid-upper arm circumference (% reference for age and sex) at baseline mean 85 (SEM 2) for patients and mean 98 (SEM 2) for controls, p<0.001. - Triceps skinfold (% reference for age and sex) at baseline mean 52 (SEM 2) for patients and mean 96 (SEM 4) for controls, p<0.001. - Standardized weight significantly improved from mean -1.23 (SEM 0.14) before to -0.70 (SEM 0.18) after supplementation in patients, p<0.05. - Standardized height significantly improved from mean -0.91 (SEM 0.28) before to -0.53 (SEM 0.38) after supplementation in patients, p<0.02. - Mid-upper arm circumference (% reference for age and sex) significantly improved from mean 85 (SEM 2) before to 91 (SEM 2) after supplementation in patients, p<0.01. - Triceps skinfold (% reference for age and sex) significantly improved from mean 52 (SEM 2) before to 63 (SEM 3) after supplementation in patients, p<0.005. - Total body potassium (g) significantly improved from mean 59.8 (SEM 6.6) before to 70.4 (SEM 8.7) after supplementation in patients, p<0.01. - Total body potassium as % reference body mass (x10^-4^) significantly improved from mean 1.68 (SEM 0.10) before to 1.95 (SEM 0.09) after supplementation in patients, p<0.01. - Muscle mass (kg) significantly improved from mean 8.21 (SEM 0.61) before to 9.69 (SEM 1.27) after supplementation in patients, p<0.01. - Muscle mass as % reference body mass significantly improved from mean 22.7 (SEM 2.3) before to 26.5 (SEM 2.3) after supplementation in patients, p<0.01. - Body mass (as percentage reference body mass) at baseline was significantly lower in patients (mean 77.7 (SEM 3.4)) compared with controls (mean 100.2 (SEM 1.5)), p<0.001. - Body fat (as percentage reference body mass) at baseline was significantly lower in patients (mean 7.9 (SEM 0.6)) compared with controls (mean 14.4 (SEM 0.5)), p<0.001. - Muscle mass (as percentage reference body mass) at baseline was significantly lower in patients (mean 22.2 (SEM 2.0)) compared with controls (mean 30.6 (SEM 1.3)), p<0.001. - Total body potassium as % reference body mass (x10^-4^) before (mean 1.68 (SEM 0.10)) and after supplementation (mean 1.95 (SEM 0.09)) in patients were not significantly different from controls (mean 1.95 (SEM 0.09)). - Muscle mass as % reference body mass before (mean 22.7 (SEM 2.3)) and after supplementation (mean 26.5 (SEM 2.3)) in patients were significantly different from controls (mean 30.6 (SEM 1.3)), p<0.01 and p<0.05 respectively. - The mean potassium content of patients was not significantly different from control values although there is an absolute deficit when expressed as a percentage reference body mass. - Patients had proportionately less fat per kilogram body mass expressed as a percentage reference body mass (and thus a larger fat-free compartment) than control values, but the muscle mass as a percentage body mass was not significantly different from control values (data not shown). - The significant improvements in weight and height (p<0.05 and p<0.01, respectively) are attributed to increments in body fat (both as a percentage actual body mass (p<0.05) and as a percentage reference body mass (p<0.01)) and muscle mass (p<0.01), including total body potassium as a percentage reference mass (p<0.01). - Total body potassium increased with a mean of 17.4% in 5 CF patients compared with a mean increase in weight of 8.7%, with individual increases in total body potassium and muscle mass in all patients, indicating lean body mass accretion.   Physical fitness outcomes and assessment:   - Clinical scores were assessed using a scoring method (not further specified).   Timing:  Before and after 6 months of supplementation.  Physical fitness results:   - Clinical scores ranged from 35 to 79 at the beginning of the study (average 68 total, 48 pulmonary, 19 general), and improved in all but one case (who eventually died). After supplementation, average scores were 74 total (p<0.01), 51 pulmonary (p<0.05), and 22 general (p<0.001).   Adverse effects outcomes and assessment:   - Clinical data, including tolerance and complications, were recorded (method not specified). Compliance was based on sachet usage.   Timing:  Before and after 6 months of supplementation.  Adverse effects results:   - All patients tolerated the food supplements reasonably well. Occasional complications were encountered with the nocturnal intragastric feeds, including vomiting, but in no case this was a persistent problem. Patients taking oral supplements usually preferred the flavoured preparation administered cold as a drink or slush, and compliance was good. | Additional remarks:  Grants from the National Health and Medical Research Council, The Australian Institute for Nuclear Science and Engineering, and the Cystic Fibrosis Association of Australia.  Selection bias:  Unclear risk  Reason: Not reported if all eligible patients were included or it was a random sample.  Attrition bias:  High risk for muscle mass and lean body mass + Low risk for all other outcomes  Reason: Muscle mass was assessed in 6/7 (85.7%) and lean body mass was assessed in 5/7 (71.4%) of patients, all other outcomes were assessed in all patients.  Detection bias:  Low risk for weight/ body mass, muscle mass and lean body mass + High risk for all other outcomes  Reason: Outcome assessors were not blinded but weight/ body mass, muscle mass and lean body mass are an objective outcomes.  Confounding:  High risk  Reason: Important prognostic factors were not taken into account in the analyses. |

Abbreviations: CF = cystic fibrosis, SD = standard deviation, SEM = standard error of mean.

| **To explore the impact of diet on growth in adolescents with Crohn disease and growth failure.** | | | | |
| --- | --- | --- | --- | --- |
| Motil et al. The effect of disease, drug, and diet on whole body protein metabolism in adolescents with Crohn disease and growth failure. The Journal of pediatrics 1982;101(3):345-51. | | | | |
| **Study characteristics** | **Patients** | **Intervention** | **Main outcomes** | **Additional remarks**  **Risk of bias assessment** |
| Study design:  Prospective intervention study. | Number and type of patients:  n=6 male adolescent patients with classic, clinically stable Crohn disease and growth failure.  Exclusion criteria:  Not reported.  Type of disease:  The site of Crohn disease (n=5 ileum, n=3 colon) and clinical and biochemical evidence of disease activity were variable.  Treatment details:  Medication during study includes prednisone (n=4, 10-30 mg (alternate) daily), sulfasalazine (n=5, 1-2 gm daily) and diphenoxylate (n=3, 1-2 tablets daily).  Follow-up duration since disease diagnosis:  Duration of Crohn disease from date of diagnosis ranged between 2-5 years.  Age at testing intervention group:  Mean 15.0 (SD 1.0) years.  Gender:  6 males (100.0%).  Controls:  n=5 healthy, age-matched controls.  Age mean 14.5 (SD 1.9) years.  5 males (100.0%). | Type of supplement:  Dietary supplement consisting of a commercially prepared formula (Osmolite or Ensure, Ross Laboratories, Columbus, Ohio).  Dose:  Estimated to provide a 40% increase in dietary protein and energy (1,500 ml).  Way of supplementation:  Administered via a silicone rubber, mercury-weighted (size 7.3 mm) nasogastric tube (Keofeed, Mountain View, Calif.) for eight to ten hours. One patient received the supplement through a feeding gastrostomy.  Frequency:  Every night.  Duration:  7 months.  Standard of care:  Not applicable. | Anthropometric and body composition outcomes and assessment:   - Anthropometric measurements, including weight, height, height for age, weight for height, arm circumference, triceps skinfold and arm muscle circumference, were obtained (method not specified). - Baseline lean body mass determined by whole body potassium measured as potassium. - Baseline muscle mass estimated from urinary creatinine excretion.   Timing:  At baseline and after 7 months for patients and 6 months after study in controls.  Anthropometric and body composition results:   - Height (cm) at baseline mean 145.3 (SD 11.0) for patients and mean 161.8 (SD 10.3) for controls, p<0.05. - Weight (kg) at baseline mean 38.2 (SD 3.7) for patients and mean 56.0 (SD 16.3) for controls, p<0.05. - Height for age (%) at baseline mean 86.3 (SD 4.2) for patients and mean 98.2 (SD 6.9) for controls, p<0.02. - Weight for height (%) at baseline mean 106.0 (SD 20.5) for patients and mean 105.1 (SD 26.1) for controls. - Arm circumference (cm) at baseline mean 23.0 (SD 2.0) for patients and mean 26.9 (SD 4.1) for controls. - Triceps skinfold (cm) at baseline mean 14.2 (SD 6.7) for patients and mean 12.9 (SD 5.9) for controls. - Arm muscle circumference (mm) at baseline mean 18.5 (SD 1.2) for patients and mean 22.9 (SD 3.3) for controls, p<0.01. - Lean body mass (kg) at baseline mean 29.9 (SEM 1.7) for patients and mean 42.7 (SEM 4.3) for controls, significant reduction compared with controls of 30%, p<0.025. - Muscle mass (kg) at baseline mean 21.8 (SEM 0.6) for patients and mean 33.8 (SEM 4.3) for controls, significant reduction compared with controls of 35%, p<0.025. - Height gain (cm/month) was five times greater after mean 7.0 (SEM 0.8) months of supplementation (mean 0.50 (SEM 0.16)) than during mean 10.0 (SEM 1.4) months prior to supplementation (mean 0.10 (SEM 0.08)), p<0.05. This rate was similar to the height velocity of controls (mean 0.38 (SEM 0.12). - Weight gain (cm/month) was nearly six times greater after mean 7.0 (SEM 0.8) months of supplementation (mean 1.22 (SEM 0.25)) than during mean 10.0 (SEM 1.4) months prior to supplementation (mean 0.21 (SEM 0.09)), p<0.05. This rate was significantly greater compared to the weight gain of the controls (mean 0.40 (SEM 0.17)), p<0.01.   Physical fitness outcomes and assessment:  Not reported.  Timing:  Not reported.  Physical fitness results:  Not reported.  Adverse effects outcomes and assessment:  Not reported.  Timing:  Not reported.  Adverse effects results:  Not reported. | Additional remarks:  No.  Selection bias:  Unclear risk  Reason: Not reported if all eligible patients were included or it was a random sample.  Attrition bias:  High risk for weight and height  Reason: Weight and height after supplementation were assessed in 4/6 (66.7%) of patients.  Detection bias:  Low risk for weight + High risk for height  Reason: Outcome assessors were not blinded but weight is an objective outcome.  Confounding:  High risk  Reason: Important prognostic factors were not taken into account in the analyses. |

Abbreviations: SD = standard deviation, SEM = standard error of mean.

| **To describe weight achievement of protein and energy-enriched (PE) formula in infants with a prolonged PICU admission.** | | | | |
| --- | --- | --- | --- | --- |
| Eveleens et al. Weight improvement with the use of protein and energy enriched nutritional formula in infants with a prolonged PICU stay. Journal of human nutrition and dietetics : the official journal of the British Dietetic Association 2019;32(1):3-10. | | | | |
| **Study characteristics** | **Patients** | **Intervention** | **Main outcomes** | **Additional remarks**  **Risk of bias assessment** |
| Study design:  Retrospective database study. | Number and type of patients:  n=70 children with a prolonged PICU stay and enteral feeding with PE-formula of ≥14 days.  Exclusion criteria:  Oral intake other than human milk or formula, interruptions from PE-formula of more than 5 days or of more than 20% of the total duration of PE-formula use and less than two weight measurements reported or weight measurements less than 14 days apart during the period of PE-formula.  Type of disease:  Primary diagnosis: n=24 cardiac surgery, n=13 respiratory (includes pneumonia, respiratory syncytial virus bronchiolitis and bronchopulmonary dysplasia), n=8 cardiac, n=4 neurology (includes neurosurgery, neurotrauma and epilepsy), n=3 GI surgery, n=1 surgery other, n=1 infection/sepsis, n=16 other.  Treatment details:  Not reported.  Follow-up duration since PICU admission:  From as soon as possible, preferably the day after admission until weaning from ventilation or when the weight goal was achieved.  Age at testing intervention group:  Median 76.2 (IQR 30.0-181.8) days. Range 37 post-menstrual weeks-12 months.  Gender:  34 females (48.6%), 36 males (51.4%).  Controls:  No. | Type of supplement:  PE-formula.  Dose:  100 kcal/100 mL; 2.6 g protein/100 mL. If tolerated, feeding was increased until an energy target of twice the individual calculated resting energy expenditure.  Way of supplementation:  n=45 post-pyloric feeding and feeding strategy n=27 continuous, n=10 portion and n=33 both.  Frequency:  Daily.  Duration:  Duration from admission to start PE-formula median 8 (IQR 1-24) days. Median duration of receiving PE-formula during PICU stay 29.2 (IQR 20.9-54.3) days. Median duration before PE-formula was stopped and switched to standard formula or an extensively hydrolysed (whey-based) protein and energy-enriched formula 24.5 (IQR 15.9-55.0) days.  Standard of care:  Not applicable. | Anthropometric and body composition outcomes and assessment:   - Weight measurements performed according to local protocol (method not specified). - Height was measured (method not specified).   Timing:  At the start and end of PE-formula use.  Anthropometric and body composition results:   - Weight at start (g) median 3943 (IQR 3289 - 5803). - WFA Z-score at start mean -1.93 (SD 1.68). - HFA Z-score at start median -1.44 (IQR -2.44 - -0.75). - WFA Z-score significantly increased during PE-formula use (mean 0.48 (SD 1.10), p<0.001). - The number of patients with WFA Z-score <-2 decreased from 33 (47%) to 23 (33%) at the end of PE-formula use. - Body weight (g/kg/day) increased during PE-formula use (overall median 5.80 (IQR 3.28 - 9.04) and in infants between the age 0–3 months (n=40) median 7.54 (IQR 4.70 - 10.47), 3–6 months (n=13) median 4.49 (IQR 1.48 - 5.82) and 6–12 months (n=17) median 3.88 (IQR 2.92 – 6.18)). - Lower WFA Z-score at start was associated with a higher increase in WFA Z-score during PE-formula use (r^2^ = 0.26; β −0.35; 95% CI = −0.50 - −0.19; p<0.001). Other predictive baseline variables (e.g. WFA Z-score at birth, respiratory diagnosis, corrected age at start and reason to start) were not associated with changes in WFA Z-score during PE-formula use.   Physical fitness outcomes and assessment:  Not reported.  Timing:  Not reported.  Physical fitness results:  Not reported.  Adverse effects outcomes and assessment:  Not reported.  Timing:  Not reported.  Adverse effects results:  Not reported. | Additional remarks:  No.  Selection bias:  Unclear risk  Reason: Unclear how many eligible patients were excluded.  Attrition bias:  Low risk for all outcomes  Reason: Retention was available in 65/70 (92.9%) of patients and all other outcomes were assessed in all patients.  Detection bias:  Low risk for weight and gastrointestinal interruptions + High risk for all other outcomes  Reason: Outcome assessors were not blinded but weight and gastrointestinal interruptions are objective outcomes.  Confounding:  High risk  Reason: Follow-up was not taken into account in the analyses, other important prognostic factors were taken into account in the analyses. |

Abbreviations: CI = confidence interval, GRV = gastric residual volume, HFA = height-for-age, IQR = interquartile range, PE = protein and energy-enriched, PICU = pediatric intensive care unit, SD = standard deviation, WFA = weight-for-age.

References

1. Leclercq E, Leeflang MM, van Dalen EC, Kremer LC. Validation of search filters for identifying pediatric studies in PubMed. *J Pediatr*. Mar 2013;162(3):629-634.e2. doi:10.1016/j.jpeds.2012.09.012

2. Mulder RL, van Dalen EC, Brown MC, Skinner R, Hudson MM, Kremer LCM. Handbook for guideline development; collaboration between International Guideline Harmonization Group and PanCare Guidelines Group. 2024

3. Grimes DA, Schulz KF. Cohort studies: marching towards outcomes. *Lancet*. Jan 26 2002;359(9303):341-5. doi:10.1016/s0140-6736(02)07500-1

4. Laupacis A, Wells G, Richardson WS, Tugwell P. Users' guides to the medical literature. V. How to use an article about prognosis. Evidence-Based Medicine Working Group. *Jama*. Jul 20 1994;272(3):234-7. doi:10.1001/jama.272.3.234
